# Screening Biocontrol Agents for Cash Crop Fusarium Wilt Based on Fusaric Acid Tolerance and Antagonistic Activity against *Fusarium oxysporum*

**DOI:** 10.3390/toxins15060381

**Published:** 2023-06-05

**Authors:** Qinggang Guo, Shixin Li, Lihong Dong, Zhenhe Su, Peipei Wang, Xiaomeng Liu, Ping Ma

**Affiliations:** Institute of Plant Protection, Hebei Academy of Agricultural and Forestry Sciences, Integrated Pest Management Innovation Centre of Hebei Province, Baoding 071000, China; qinggangguo77@haafs.org (Q.G.); lishixin@haafs.org (S.L.); lihongdong@haafs.org (L.D.); suzhenhe@haafs.org (Z.S.); peipeiwang@haafs.org (P.W.); liuxiaomeng@haafs.org (X.L.)

**Keywords:** *Bacillus velezensis*, biological control, Fusarium wilt, fusaric acid, tolerant

## Abstract

Fusarium wilt, caused by *Fusarium oxysporum*, is one of the most notorious diseases of cash crops. The use of microbial fungicides is an effective measure for controlling Fusarium wilt, and the genus *Bacillus* is an important resource for the development of microbial fungicides. Fusaric acid (FA) produced by *F. oxysporum* can inhibit the growth of *Bacillus*, thus affecting the control efficacy of microbial fungicides. Therefore, screening FA-tolerant biocontrol *Bacillus* may help to improve the biocontrol effect on Fusarium wilt. In this study, a method for screening biocontrol agents against Fusarium wilt was established based on tolerance to FA and antagonism against *F. oxysporum*. Three promising biocontrol bacteria, named B31, F68, and 30833, were obtained to successfully control tomato, watermelon, and cucumber Fusarium wilt. Strains B31, F68, and 30833 were identified as *B. velezensis* by phylogenetic analysis of the 16S rDNA, *gyrB*, *rpoB,* and *rpoC* gene sequences. Coculture assays revealed that strains B31, F68, and 30833 showed increased tolerance to *F. oxysporum* and its metabolites compared with *B. velezensis* strain FZB42. Further experiments confirmed that 10 µg/mL FA completely inhibited the growth of strain FZB42, while strains B31, F68, and 30833 maintained normal growth at 20 µg/mL FA and partial growth at 40 µg/mL FA. Compared with strain FZB42, strains B31, F68, and 30833 exhibited significantly greater tolerance to FA.

## 1. Introduction

Fusarium wilt, caused by different *Fusarium oxysporum* special forms, is one of the most notorious diseases of cash crops throughout the world [1]. Grafting has been successfully applied to control Fusarium wilt in cucumber and watermelon [2,3]. However, grafting not only increases production costs but also reduces the quality of fruits. The pathogen occurs in soil, so the chemicals used to suppress Fusarium wilt are inefficient and may pollute soil and groundwater when excessively used. In addition, it is also difficult to control this disease by crop rotation due to the survival of *F. oxysporum* in the soil for long periods of time [4]. Therefore, it is necessary to explore new measures for controlling Fusarium wilt. Microbial fungicides have been confirmed as effective and environmentally friendly measures to control crop soilborne diseases. *Pseudomonas spp*. demonstrated a significant reduction in disease incidence and an increase in chickpea growth [5,6]. Meanwhile, *Trichoderma* spp. effectively suppressed Fusarium wilt through competition for nutrients and space, mycoparasitism, antibiosis, and improved plant growth, leading to showing well-controlled efficiency [7,8,9]. *Bacillus subtilis* and its closely related species are important resources for developing microbial fungicides mainly due to the formation of heat-resistant spores, which are conducive to product development and shelf life extension [10,11]. In recent years, *Bacillus* strains have been developed as promising biocontrol agents for suppressing Fusarium wilt [12]

The main mechanisms by which *B. subtilis* suppresses plant soilborne diseases involve inhibiting the growth of phytopathogens by producing antibiotics [13,14], competing for nutrients and niches with phytopathogens via efficient colonization of plant rhizospheres [15], and inducing systemically acquired plant resistance [16,17]. However, effective colonization of plant rhizospheres and maintenance of high populations in biocontrol agents are prerequisites for successfully suppressing soilborne diseases [18]. The rhizosphere colonization of *Bacillus* is not only related to its own genetic factors (such as biofilm formation and chemotaxis abilities) [13] but also affected by biotic and abiotic factors in rhizosphere soil [19].

Fusaric acid (FA) is a mycotoxin produced by *Fusarium* species that can act synergistically with other Fusarium toxins [20]. FA plays a major role in causing plant wilt and death [21]. In addition, FA also showed inhibitory abilities against the growth of various microorganisms, including rhizosphere bacteria, especially *Bacillus* species [22]. Therefore, the existence of massive amounts of *F. oxysporum* in the plant rhizosphere could inhibit the growth and colonization of biocontrol bacteria in the rhizosphere via FA production, thereby decreasing the biocontrol efficacy of biocontrol bacteria against Fusarium wilt [23]. In addition, FA also inhibited the production of antifungal active compounds, such as the phenazine-1-carboxamide (PCN), produced by *Pseudomonas chlororaphis* [24], and 2,4-diacetylphloroglucinol (2,4-DAPG), produced by *P. fluorescens* [25]. Enhancing FA tolerance may improve the biocontrol efficacy of biocontrol bacteria against Fusarium wilt. Hideyoshi and Ryutaro found that FA inhibited the growth of *P. solanacearum*; however, a mutant of *P. solanacearum* could degrade FA and showed resistance to it, so the mutant strain increased the biological control efficacy against tomato Fusarium wilt [26]. Simonetti et al. screened bacteria that could use FA as the sole carbon and nitrogen source and identified a strain of *Burkholderia ambifaria* that could successfully suppress barley wilt [27]. Therefore, screening of *Bacillus* strains resistant to FA and able to inhibit the growth of *F. oxysporum* is expected to yield promising biocontrol agents for crop Fusarium wilt.

In this study, a method for screening *Bacillus* isolates with biocontrol abilities for cash crop Fusarium wilt was developed, and three *Bacillus* strains were obtained that could successfully suppress Fusarium wilt in cucumber, tomato, and watermelon. Additionally, the taxonomy of the three *Bacillus* strains was clarified by joint analysis of multiple gene sequences. The tolerance of the three *Bacillus* strains to *F. oxysporum* and its metabolites, as well as FA, were evaluated by coculture assays.

## 2. Results

### 2.1. Screening of Bacteria with Tolerance to FA and Antagonistic Abilities against Foc

A total of 238 bacteria with tolerance to FA (20 µg/mL) were obtained from cucumber and tomato rhizosphere soil, among which 68 isolates showed stronger antagonistic abilities (inhibitory zone ≥ 0.5 cm) against the growth of Foc in vitro. Information on the 68 isolates is listed in Supplementary Table S1.

### 2.2. Screening of Biocontrol Agents against Cucumber Fusarium Wilt

The biocontrol effects of the 68 antagonistic bacteria on cucumber Fusarium wilt were evaluated by pot experiments. The results showed that five antagonistic bacteria could effectively suppress cucumber Fusarium wilt. Among them, strain B31 had the strongest biocontrol ability, and 97% control efficacy was achieved against cucumber Fusarium wilt. Followed by strains 30833 and F38, the biocontrol efficacies were 88.02% and 82.69%, respectively. The biocontrol efficacies of strains F68 and A84 against cucumber Fusarium wilt were 74.04% and 61.54%, respectively (Table 1).

### 2.3. Biocontrol Effects of Biocontrol Agents on Fusarium Wilt of Tomato and Watermelon

The biocontrol effects of strains B31, 30833, F68, F38, and A84 on the Fusarium wilt of tomato and watermelon were further evaluated by pot experiments. The results showed that all five biocontrol agents could successfully suppress tomato Fusarium wilt (Table 1), with strains B31 and 30833 having the best biocontrol effects, achieving biocontrol efficacies of 85.7%. They were followed by strains F38 and F68, with biocontrol efficacies of 83.3%. Biocontrol efficacy of 71.4% was obtained by strain A84 against tomato Fusarium wilt. Four biocontrol agents effectively suppressed watermelon Fusarium wilt, among which strain B31 had the strongest biocontrol effect, achieving a biocontrol efficacy of 84.26%. Biocontrol efficacies of 65.22%, 64.13%, and 61.96% were achieved by strains 30833, F68, and F38, respectively. Only 27.8% biocontrol efficacy for watermelon fusarium wilt was obtained by strain A84. Taking the results together, strains B31, F68, and 30833 showed stronger biocontrol abilities against Fusarium wilt in cucumber, tomato, and watermelon.

### 2.4. Identification of Strains B31, F68, and 30833

**Figure 1:**
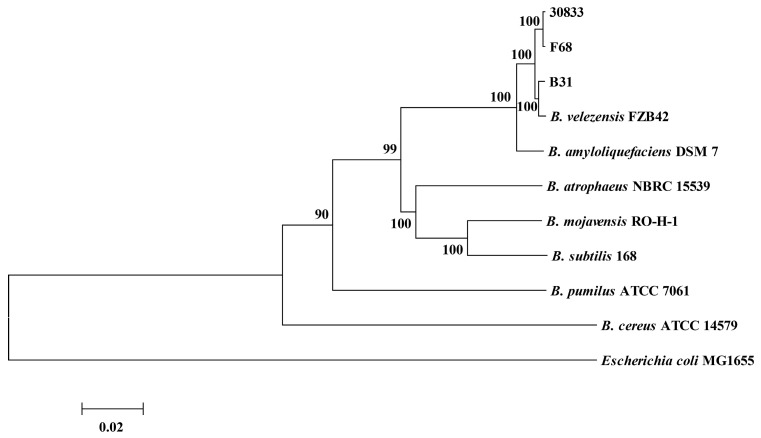
Phylogenetic tree generated from analysis of multigene genealogies of *gyrB*, *rpoB,* and *rpoC*. The scale bar represents a phylogenetic distance of 0.02. The numbers on each node represent the support of 1000 bootstrap replicates; only bootstrap values >80% are shown. Branches represent the evolutionary lineages that lead to the different species and/or strains.

The BLAST analysis of the 16S rDNA sequences of strains B31, F68, and 30833 revealed significant identity (>99%) with *Bacillus subtilis* 168 and *B. velezensis* FZB42, indicating that strains B31, F68, and 30833 belonged to the family of *B. subtilis*. The housekeeping genes *gyrB*, *rpoB,* and *rpoC* were further amplified from strains B31, F68, and 30833 (GenBank accession codes are provided in Supplementary Table S2), and the phylogenetic tree was constructed based on the aligned sequences of *gyrB*, *rpoB,* and *rpoC*. The results showed that strains B31, F68, and 30833 were clustered together with *B. velezensis* strain FZB42 (Figure 1). Therefore, the results confirmed that strains B31, F68, and 30833 belonged to the species *B. velezensis*.

### 2.5. Strains B31, F68, and 30833 Increased Tolerance to the Inhibitory Effect of Foc

**Figure 2:**
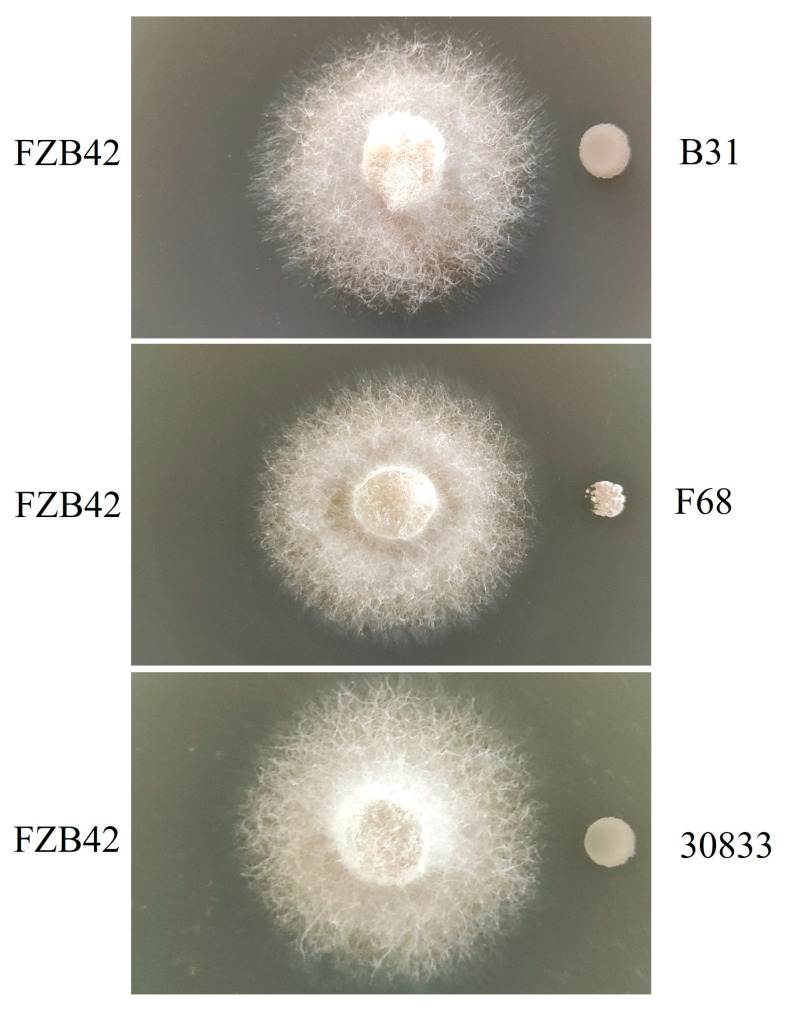
Inhibitory abilities of *Fusarium oxysporum* against the growth of *Bacillus velezensis* strains B31, F68, 30833, and FZB42.

The antagonistic activities of Foc against the growth of *B. velezensis* strains B31, F68, 30833, and *B. velezensis* strain FZB42 were tested, and the results showed that Foc could inhibit the growth of strain FZB42, as revealed by its inability to form colonies when cocultured with Foc. However, the colonies of *B. velezensis* strains B31, F68, and 30833 grew normally, and the side close to Foc did not show significant growth inhibition (Figure 2).

### 2.6. Strains B31, F68, and 30833 Showed Increased Tolerance to Foc Metabolites

**Figure 3:**
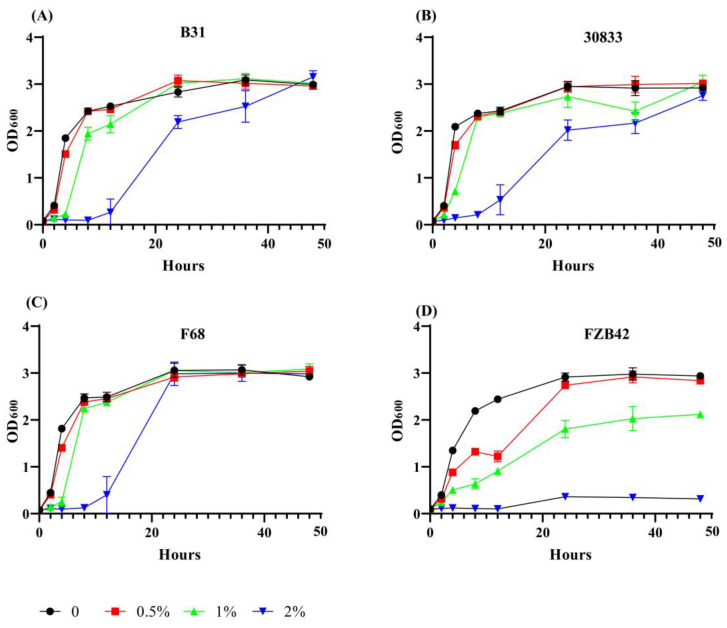
Tolerance to the metabolites of *Fusarium oxysporum* f. sp. *cucumerinum* (Foc) by *Bacillus velezensis* strains B31 (**A**), 30833 (**B**), F68 (**C**), and FZB42 (**D**). The solid line with black circles, red squares, green up-triangles, and blue down-triangles represent bacteria cultured in LB medium, LB medium supplied with 0.5% cell-free metabolites of Foc, LB medium supplied with 1% cell-free metabolites of Foc, LB medium supplied with 2% cell-free metabolites of Foc, respectively.

The effects of Foc metabolites on the growth of *B. velezensis* strains B31, F68, 30833, and FZB42 were compared (Figure 3). When cultured in LB medium, all of the bacteria showed similar growth rates. When cultured in LB medium supplied with 0.5% Foc metabolites, the growth of strain FZB42 was partially inhibited, and the cell density (OD_600_) value was 1.22 and 2.74 at 12 HAI and 24 HAI, respectively. When LB medium was supplied with 1.0% metabolites, the OD_600_ of the FZB42 suspension was 0.91 and 1.81 at 12 HAI and 24 HAI, respectively. The growth of strain FZB42 was almost completely inhibited when LB medium was supplied with 2% Foc metabolites. Compared with strain FZB42, *B. velezensis* strains B31, F68, and 30833 showed increased tolerance to Foc metabolites. Supplementation with 0.5% and 1% Foc metabolites had no significant effect on the growth of strains B31, F68, and 30833. Supplementation with 2% Foc metabolites partially inhibited the growth of strains B31, F68, and 30833, and the OD_600_ values were 0.27, 0.40, and 0.53 at 12 HAI, respectively. Then, strains B31, F68, and 30833 grew rapidly, and the OD_600_ values were 2.19, 2.99, and 2.02 at 24 HAI, respectively.

### 2.7. Tolerance of Strains B31, 30833, and F68 to FA

**Figure 4:**
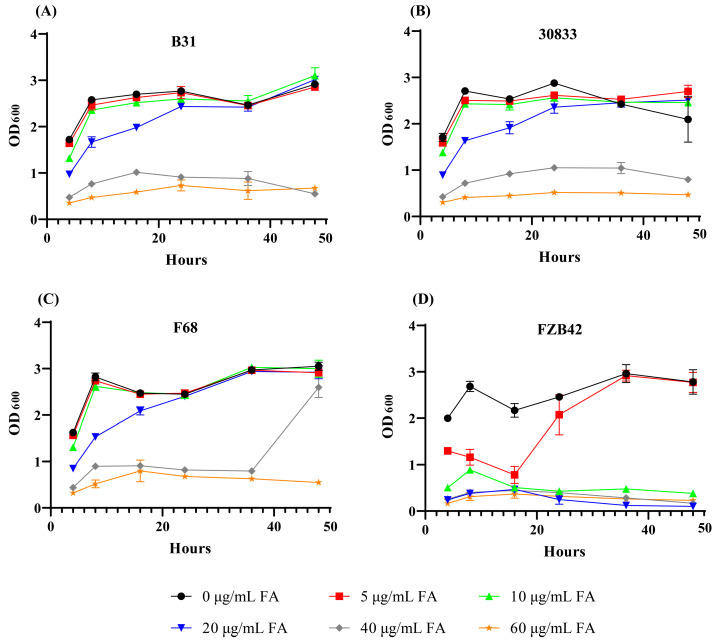
Tolerance to fusaric acid (FA) of *Bacillus velezensis* strains B31 (**A**), 30833 (**B**), F68 (**C**), and FZB42 (**D**). The solid line with black circles, red squares, green up-triangles, blue down-triangles, grey squares, and orange pentagrams represent bacteria cultured in LB medium, LB medium supplied with 5 µg/mL FA, 10 µg/mL FA, 20 µg/mL FA, 40 µg/mL FA, and 60 µg/mL FA, respectively.

The tolerance of *B. velezensis* strains B31, F68, 30833, and FZB42 to FA was compared in LB medium supplied with different concentrations of FA (Figure 4). When cultured in LB medium, all of the bacteria showed similar growth rates (OD_600_). When LB medium was supplied with 5 µg/mL FA, the growth of strain FZB42 was partially inhibited, and the OD_600_ was 0.78 at 16 HPI. When LB medium was supplied with 10 µg/mL FA, the growth of strain FZB42 was almost completely inhibited. LB medium supplemented with 5 and 10 µg/mL FA did not affect the growth of strains B31, F68, and 30833. LB medium supplied with 20 µg/mL FA only slightly decreased the growth of strains B31, F68, and 30833, and the OD_600_ values were 1.76, 2.05, and 1.90 at 16 HPI, respectively. Strains B31, F68, and 30833 maintained low concentrations even at 40 µg/mL FA, and the OD_600_ values were 1.0, 0.91, and 0.95 at 16 HAI, respectively.

## 3. Discussion

The purpose of this study was to screen biocontrol agents for crop Fusarium wilt from rhizosphere soil. The genus *Bacillus* is an important biocontrol resource for developing microbial fungicides, mainly due to its varied biocontrol mechanisms and formation of heat-resistant spores [29,30]. In the soil, *Bacillus* mostly occurs in the form of spores, which are generally tolerant to high temperatures [31,32]. Therefore, the soil aqueous solutions were first treated at 80 °C for 10 min to remove most of the high-temperature-sensitive bacteria and retain *Bacillus*. Effective colonization of bacteria in the plant rhizosphere and maintenance of a high population are the prerequisites for developing biocontrol effects. The population of bacteria in the plant rhizosphere is highly affected by environmental factors, in particular, the microecological environment [33]. Complex microbial structures are recruited by root exudates to the plant rhizosphere, including beneficial bacteria and plant pathogens [34]. *Fusarium* is a dominant soilborne fungus, and some specialized forms can infect plants and cause Fusarium wilt [35]. Fusaric acid (FA) produced by *Fusarium* not only causes plant wilt but also inhibits the growth of bacteria [36,37,38]; comparatively, *Bacillus* is more sensitive to FA than other genera. Therefore, *Fusarium* in the plant rhizosphere can affect the colonization of *Bacillus* and thus decrease its biocontrol effect on Fusarium wilt [39]. FA can inhibit the growth of different genera of bacteria; however, a strain of *Stenotrophomonas maltophilia* could resist FA via an inducible tripartite efflux pump, FuaABC [40]. In addition, an FA-resistance gene cluster (*fusA*, *B*, *C*, *D,* and *E*) was also cloned from *P. cepacia* and conferred resistance to 500 μg/mL FA [41]. Therefore, it is expected that *Bacillus* strains with resistance to FA, as well as antagonism to *Fusarium,* can be obtained. Bacon et al. tested the inhibitory abilities of FA against different strains of *B. mojavensis*. The results showed that 66 µM (approximately 12 µg/mL) FA could inhibit the growth of most strains by 78% [37]. In the present study, the soil samples were spread on plates supplied with 20 μg/mL FA, and a total of 238 isolates with vigorous growth were obtained, which were preliminarily identified as *Bacillus* according to colony morphology. By antifungal activity tests, 68 *Bacillus* strains with strong inhibitory ability against *F. oxysporum* were obtained. Ultimately, three *Bacillus* strains with excellent biocontrol effects against Fusarium wilt of cucumber, watermelon, and tomato were obtained. Therefore, the screening system developed in this study was able to rapidly obtain biocontrol *Bacillus* with an effective ability to suppress crop Fusarium wilt.

It was expected that FA-tolerant bacteria would have increased resistance to the metabolites of Fusarium. In this study, the interaction between *Bacillus* and *F. oxysporum* was tested. The results showed that *F. oxysporum* significantly inhibited the growth of strain FZB42, while the FA-tolerant strains B31, F68, and 30833 showed increased tolerance to *F. oxysporum*. It was further demonstrated by coculture experiments that adding *F. oxysporum* cell-free fermentation broth to the culture could inhibit the growth of strain FZB42, but strains B31, F68, and 30833 were able to tolerate the cell-free fermentation broth.

In addition to FA, *F. oxysporum* also produces other mycotoxins, such as beauvericin, fumonisin, enniatins, and zearalenone [37]. At present, no report has shown that other mycotoxins can inhibit the growth of *Bacillus*. Therefore, we compared the tolerance to FA between strains B31, F68, 30833, and strain FZB42. The results showed that 5 µg/mL FA could partially inhibit the growth of FZB42 and that 10 µg/mL FA could completely inhibit the growth of FZB42; however, strains B31, F68, and 30833 were able to grow normally under 20 µg/mL FA, and partially growth under 40 µg/mL FA. According to the above results, we speculated that the FA-tolerant strains could grow normally when interacting with *F. oxysporum* and inhibit the growth of *F. oxysporum* by producing antifungal active compounds, mainly lipopeptide antibiotics such as fengycin and iturins [13], thus exerting a biological control effect against Fusarium wilt.

*B. subtilis* and its closely related species, including *B. subtilis*, *B. velezensis*, *B. amyloliquefaciens*, *B. mojavensis,* and *B. atrophaeus*, exhibit high phenotypic and genetic similarities. Therefore, it is difficult to exactly identify the species in the *B. subtilis* family by physiology, biochemistry, and 16S rDNA sequence analysis [42]. At present, the joint analysis of multiple housekeeping gene sequences is used to identify and discriminate strains belonging to the *B. subtilis* family [42]. In this study, the three FA-tolerant bacteria with biocontrol abilities against Fusarium wilt of important economic crops were identified as *B. velezensis* by joint analysis of the sequences of *gyrB*, *rpoB,* and *rpoC*, but the gene sequences of the three bacteria were different, indicating that they were not the same strain. Strain FZB42 is a representative strain of *B. velezensis*; however, strain FZB42 was sensitive to the metabolites of Foc as well as to FA; therefore, tolerance to FA was not a characteristic of the species of *B. velezensis*. Ruiz et al. reported that the chelation of Fe^3+^ by FA was one of the mechanisms through which FA inhibits the growth of *P. protegens* [43]. Our previous study showed that supplementing excess Fe^3+^ in the culture medium did not decrease the inhibitory ability of FA on the growth of strains B31, F68, 30833, and FZB42, so the inhibitory ability of FA on the growth of *B. velezensis* was not associated with chelation of Fe^3+^. Therefore, the mechanism by which strains B31, F68, and 30833 tolerate FA should be clarified in future research.

## 4. Conclusions

This study established a method for screening biocontrol agents against Fusarium wilt based on tolerance to FA and antagonism against *F. oxysporum*. Three promising biocontrol bacteria, named B31, F68, and 30833, were obtained to successfully control tomato, watermelon, and cucumber Fusarium wilt. *B. velezensis* strains B31, F68, and 30833 showed increased tolerance to *F. oxysporum* and its metabolites compared with *B. velezensis* strain FZB42. 10 µg/mL FA completely inhibited the growth of FZB42, while strains B31, F68, and 30833 maintained normal growth at 20 µg/mL FA and partial growth at 40 µg/mL FA. Compared with strain FZB42, strains B31, F68, and 30833 exhibited significantly greater tolerance to FA. Our results established a new method to screen biocontrol agents for crop Fusarium wilt from rhizosphere soil.

## 5. Materials and Methods

### 5.1. Screening of Bacillus Strains with FA Tolerance

One gram of soil collected from healthy cucumber and tomato rhizospheres was suspended in 100 mL of sterile water, incubated at 80 °C for 10 min, and then serially diluted. One hundred microliters of each dilution were spread on LB agar plates supplemented with 20 μg/mL FA and cultured at 30 °C for 24 h. Bacteria with vigorous growth were selected for further antifungal activity tests.

### 5.2. Antifungal Activity of Bacillus

The inhibitory abilities of *Bacillus* isolates against the growth of *F. oxysporum* f. sp. *cucumerinum* (Foc) were tested by the double-culture method. A 6-mm-diameter plug of Foc was inoculated on the center of a PDA plate, and isolates of *Bacillus* were inoculated at four peripheral sites at a distance of 2.5 mm from the Foc plug. After incubation at 25 °C for 5 days, the isolates with strong antagonistic activity against the growth of Foc were selected according to the size of the inhibition zone.

### 5.3. Determination of the Control Effect of Bacillus on Cucumber Wilt, Tomato Wilt, and Watermelon Wilt

Antagonistic bacteria were inoculated in 200 mL LB medium and cultured at 37 °C and 180 rpm for 24 h. The fermentation was centrifuged at 10,000 rpm for 20 min, and the cells were collected and adjusted to 10^8^ CFU/mL with sterile water. Cucumber seeds (Zhongnong No. 6, susceptible to Fusarium wilt), tomato (“Namei”, susceptible to Fusarium wilt), and watermelon (“Zaojia” susceptible to Fusarium wilt) were surface-sterilized and germinated at 25 °C. The germinated seeds were transplanted into seedling trays filled with sterile vermiculite. When the cotyledons were fully expanded, 3 mL of bacterial solution was directly applied to the root of each cucumber seedling, tomato seedling, and watermelon seedling, respectively. Three days after treatment, the seedlings were gently removed from the vermiculite and transplanted into plastic pots (10 × 10 cm) filled with soil that was premixed with *F. oxysporum* to a concentration of 5 × 10^5^ conidia/g soil. Two days after transplanting, another 3 mL of bacterial solution (10^8^ CFU/mL) was applied again to the root. Three replicates were performed per strain, and 9 seedlings were used per replicate. The seedlings were cultured in the greenhouse (16 h of light, 30 °C during the day, and 20 °C during the night), and seedlings treated with water were used as a blank control. The disease index (DI) was investigated 16 days after transplanting. The DI was scored on a scale from 0 to 4 according to the method reported by Zhuang et al. (2005), and it was calculated as DI = [100 × ∑ (number of diseased plants × corresponding disease rating)]/(total number × 4) [28,44]. The biocontrol efficacy of *Bacillus* isolates against Fusarium wilt was calculated based on the DI as biocontrol efficacy (%) = [(DI of control − DI of different treatments)/DI of control] × 100.

### 5.4. Identification of Biocontrol Agents

The genomic DNA of bacteria was extracted using a DNA extraction kit for bacteria (Sangon Biotech, Shanghai, China), and a partial 16S rDNA fragment was amplified using the primer set 27F/1492R [45,46]. After sequencing, the taxonomic position of bacteria at the genus level was preliminarily determined by sequence BLAST search in the National Center for Biotechnology Information (NCBI) database. Partial fragments of the housekeeping genes *gyrB*, *rpoB,* and *rpoC* in bacteria were amplified using the primers listed in Table 1. The DNA fragments were ligated into the pUC18 vector and sequenced with the primers M13F/M13R. All the DNA was sequenced by Sangon Biotech, and the sequences of *gyrB*, *rpoB,* and *rpoC* were aligned using the Clustal program [47]. The phylogenetic tree was constructed using the neighbor-joining algorithm and maximum likelihood analyses, with bootstrap values calculated from 1000 replicate runs using the routines included in MEGA software [48].

### 5.5. Antagonistic Ability of Fusarium Oxysporum against the Growth of Bacillus Strains

The antagonistic abilities of Foc against the growth of the biocontrol bacterial strains B31, F68, and 30833 were evaluated by the dual-culture method [49], and the *B. velezensis* strain FZB42 was used as a control. A 6-mm-diameter agar plug containing 5-day-old actively growing mycelia of Foc was placed on the center of a PDA plate, and 2 µL of bacterial suspension (10^8^ CFU/mL) was inoculated at a position 2.0 cm away from the edge of the fungal plug in a criss-cross direction. The antagonistic ability of Foc against bacteria was observed 24 h after inoculation.

### 5.6. Inhibitory Ability of Foc Fermentation against the Growth of Biocontrol Bacteria

Four agar plugs (Φ = 6 mm) containing 5-day-old actively growing mycelia of Foc were inoculated in 200 mL PDB medium and cultured at 25 °C and 180 rpm for 5 days. The fermentation was filtered through four layers of sterilized gauze, and the filtrate was centrifuged at 4 °C and 12,000 rpm for 20 min. The supernatant was concentrated to 10 mL in a rotary evaporator at 55 °C. After filtration through a 0.22-μM filter, the filtrate was added to 10 mL LB medium at proportions of 0, 0.5%, 1%, and 2% (*v*/*v*), and then the medium was inoculated with bacterial suspensions of strains B31, F68, 30833, and FZB42 (10^8^ CFU/mL) at a proportion of 1% (*v*/*v*). Each strain was repeated four times, and the absorbance value (OD_600_) was measured at 0, 2, 4, 8, 12, 24, 36, and 48 h after inoculation (HAI).

### 5.7. Tolerance of Biocontrol Bacteria to FA

*Bacillus* strains B31, F68, 30833, and FZB42 were inoculated in 5 mL LB medium and cultured at 37 °C and 180 rpm for 12 h. Then, 100 µL of the bacterial culture was transferred to 10 mL of LB medium supplemented with FA to final concentrations of 0, 5, 10, 20, and 40 μg/mL. Each strain was repeated four times, and the absorbance value (OD_600_) of the bacterial solution was measured at 0, 4, 8, 16, 24, 36, and 48 HAI.

### 5.8. Statistical Analysis

Statistically significant differences (*p* < 0.05) were evaluated by one-way analysis of variance (ANOVA) using SPSS 17.0. Graphs were generated by Origin 8.0 software.

## Figures and Tables

**Table 1:** Biocontrol efficacies of bacteria against Fusarium wilt of cucumber, tomato, and watermelon.

Strain	Biocontrol Efficacy (%)		
	Cucumber Fusarium Wilt	Tomato Fusarium Wilt	Watermelon Fusarium Wilt
B31	97.12 ± 0 a	85.72 ± 10.10 a	84.26 ± 10.63 a
30833	88.02 ± 2.72 ab	85.72 ± 20.20 a	65.22 ± 7.68 a
F68	74.04 ± 8.16 bc	83.33 ± 13.68 a	64.13 ± 2.66 a
F38	82.69 ± 7.07 ab	83.33 ± 23.57 a	61.96 ± 7.04 a
A84	61.54 ± 7.57 c	71.43 ± 15.71 a	27.80 ± 15.43 b

Note: The disease index (DI) = [100 × ∑ (number of diseased plants × corresponding disease rating)]/(total number × 4) [28]. The biocontrol efficacy (%) = [(DI of control − DI of different treatments)/DI of control] × 100. The lowercase letters indicate statistical significance based on one-way ANOVA with Tukey’s multiple range test using DPS software between different treatments.

## Data Availability

All the research data have been included in the manuscript.

## Supplementary Materials

The following supporting information can be downloaded at: https://www.mdpi.com/article/10.3390/toxins15060381/s1, Figure S1: Photos of pathogenicity or biocontrol tests; Table S1: Screening of antagnistic bacteria to *Fusarium oxysporum* f.sp. *cucumerinum* and tolerant to fusaric acid; Table S2: GenBank accession numbers of housekeeping genes in biocontrol agents.
