# Supplementary material for: Screening Biocontrol Agents for Cash Crop Fusarium Wilt Based on Fusaric Acid Tolerance and Antagonistic Activity against Fusarium oxysporum
Source: Toxins (Basel). 2023 Jun 5;15(6):381. doi: 10.3390/toxins15060381 (PMC10302455; doi:10.3390/toxins15060381)
Supplement: Supplementary file 1 [file toxins-15-00381-s001.zip › toxins-2379023-supplementary.pdf]

Table S1. Screening of antagnistic bacteria to *Fusarium oxysporum* f.sp. *Cucumerinum* and tolerant to fusaric acid.

Serial number	Strains	Tolerance to FA	Inhibition zone (cm）
1	FQ-32	yes	0.7
2	HMB-27544	yes	0.5
3	HMB-27248	yes	0.5
4	HMB-26390	yes	0.5
5	H-8	yes	0.6
6	H-7	yes	0.5
7	H-54	yes	0.6
8	H-39	yes	0.6
9	H-29	yes	0.6
10	H-17	yes	0.7
11	G-64	yes	0.5
12	G-60	yes	0.5
13	F-68	yes	0.5
14	F-38	yes	0.5
15	F-3	yes	0.8
16	D92	yes	0.5
17	D87	yes	0.7
18	D72	yes	0.5
19	D49	yes	0.5
20	D38	yes	0.5
21	C147	yes	0.5
22	B64	yes	0.5
23	B33	yes	0.5
24	B31	yes	0.5
25	B25	yes	0.5
26	B20	yes	0.5
27	B19	yes	0.5
28	B17	yes	0.5
29	A84	yes	0.5
30	2190035-2	yes	0.6
31	2190028-58	yes	0.5
32	2190025-60	yes	0.6
33	2190025-6	yes	0.6
34	2190025-41	yes	0.6
35	2190025-4	yes	0.5
36	2190021-10	yes	0.6
37	2190010-28	yes	0.5
38	37647	yes	0.5
39	37085	yes	0.6
40	36224	yes	0.6
41	35055	yes	0.6
42	32972	yes	0.5
43	32788	yes	0.5
44	32462	yes	0.5
45	31059	yes	0.7
46	30984	yes	0.5
47	30844	yes	0.7
48	30833	yes	0.5
49	30679	yes	0.5
50	30620	yes	0.6
51	30587	yes	0.5
52	30572	yes	0.5
53	29244	yes	0.5
54	29124	yes	0.5
55	28933	yes	0.6
56	28887	yes	0.5
57	28534	yes	0.8

58	28462	yes	0.5
59	28439	yes	0.6
60	28295	yes	0.7
61	27719	yes	0.5
62	27643	yes	0.5
63	27638	yes	0.5
64	27634	yes	0.6
65	27456	yes	0.5
66	27442	yes	0.5
67	26689	yes	0.6
68	26674	yes	0.5

Table S2. GenBank accession numbers of housekeeping genes in biocontrol agents.

**Strains**	**GenBank accession number**			
	**16S rDNA**	***gyrB***	***rpoB***	***rpoC***
B31	OP740993	OP717077	OP717080	OP717083
30833	OP740992	OP717075	OP717078	OP717081
F68	OP740991	OP717076	OP717079	OP717082

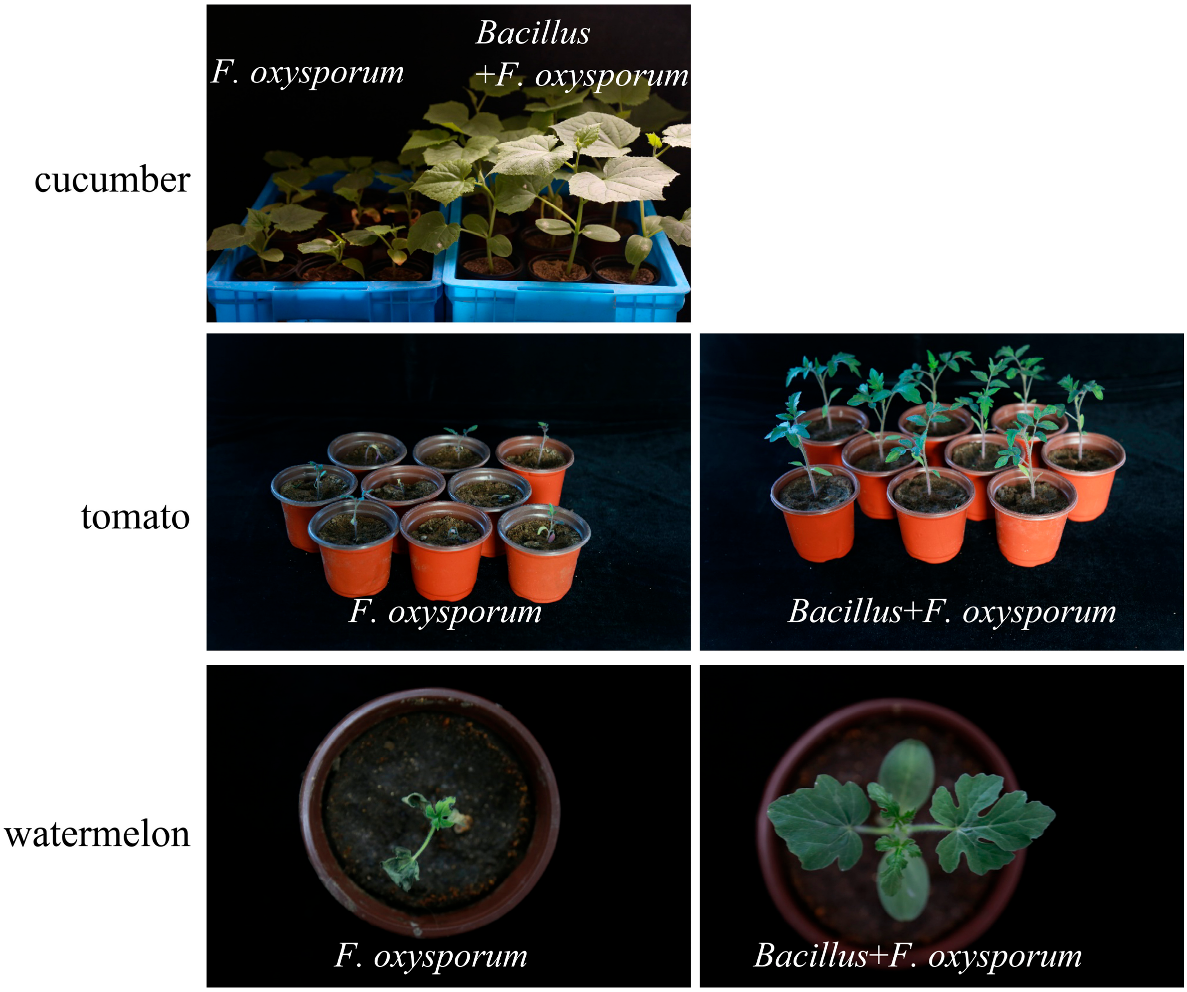

Figure S1. Photos of pathogenicity or biocontrol tests.
